# Knowledge, Attitude, and Practice of Physiotherapists in COVID-19 ICUs: A National Survey

**DOI:** 10.1155/2024/9918558

**Published:** 2024-01-17

**Authors:** Nikitha Suresh Babu, Vijay Pratap Singh, K. Shyam Krishnan, Dattatray Prabhu

**Affiliations:** ^1^Department of Physiotherapy, Kasturba Medical College, Manipal Academy of Higher Education, Mangalore, India; ^2^Department of Anesthesia, Kasturba Medical College, Manipal Academy of Higher Education, Mangalore, India

## Abstract

**Background:**

COVID-19 belongs to the beta-corona cluster that spreads enormously via aerosols. Physiotherapists must be knowledgeable about the symptoms, mode of transmission, risk mitigation strategies, and practice guidelines for COVID-19.

**Objective:**

This study aimed to assess physiotherapists' knowledge of COVID-19 guidelines, their attitude toward this new evolving field, and their practice routines in India's COVID-19 ICUs.

**Methods:**

It was a cross-sectional study. A total of 600 questionnaires were distributed through e-mail and WhatsApp to physiotherapists using Google Forms between February 2022 and January 2023. The questionnaires consisted of demographics and 23 questions in three sections about the knowledge, attitude, and practice of physiotherapists working in the COVID-19 ICU. Data analysis was carried out using Jamovi.

**Results:**

A total of 136 responses were obtained from 18 states of India. Of 136 participants, 89 were female (65.4%) and 47 were male (34.6%). The highest level of qualification was BPT (*n* = 69 (50.7%)), followed by MPT (*n* = 62 (45.6%)) and Ph.D. (3 (3.7%)). The knowledge about COVID-19 guidelines is fair. Only 21.3% of the physiotherapists received training before being deployed in COVID-19 ICUs, and the CARP protocol was well known by only as few as 10.3%. The criteria advised for close monitoring of patients during treatment was aware by 29.4%. Most physiotherapists have a good attitude toward treating COVID-19 patients; 70.63% strongly agree that physiotherapy is vital in these patients despite the risk of self-exposure, and 64.7% agree that physiotherapy should be initiated during all phases of COVID-19. Physiotherapists follow good practices for COVID-19 patients in the ICU, which is as per the guideline recommendation.

**Conclusion:**

Physiotherapists working in COVID-19 ICUs have a fair knowledge of the existing physiotherapy guidelines for COVID-19, and they exhibit good attitudes and practice patterns.

## 1. Introduction

COVID-19 (coronavirus) belongs to the beta-corona cluster that spreads enormously via aerosols [1]. It reaches the lung and the angiotensin-converting enzyme (ACE-2), seen in type I and II alveolar cells [2]. Normally, alveolar cells synthesize and secrete surfactants, implement the xenobiotic mechanism, aid water movement across the epithelium, and regenerate the alveolar epithelium postlung injury [3]. When the cells are damaged, all these functions will be affected, causing respiratory problems, other systemic manifestations, and even death.

Fever, cough, myalgia or fatigue, headache, rhinorrhea, sneezing, sore throat, gastrointestinal symptoms, pneumonia, and complicated dyspnea are some of the clinical presentations of COVID-19. Symptoms may advance from mild to severe during a week or more; deterioration may be sudden and disastrous [4, 5]. The first case of COVID-19 was reported in China in December 2019, and since then, the virus has spread rapidly. On 30^th^ January 2020, the World Health Organisation (WHO) announced it was a public health emergency of international concern [1, 6]. As of 2^nd^ December 2021, globally, more than 263 million people have tested positive [7]. The knowledge of this evolving disease is incomplete. Coronaviruses often mutate and recombine, thus challenging our understanding and clinical management of the disease. All viruses change over time, and these changes may or may not affect their properties, such as severity, speed of spread, therapeutic medicine, and diagnostic tools. Omicron and its subvariants are the recent COVID-19 variants [8]. Infection severity, vaccine effectiveness, and available treatments are still being evaluated [5]. This global pandemic has brought the world to a standstill, leading to morbidity and mortality [9].

Given the newness and urgency of this pandemic, the significance of rehabilitative care has increased due to its potential impact on mortality, morbidity, duration of ventilation, and length of ICU/hospital stay [10]. The presence of physiotherapists in the ICU contributes to the early recovery of the patients. All physiotherapeutic approaches require close contact and are considered to generate aerosols, raising safety concerns for therapists [11]. Appropriate knowledge and use of personal protection equipment (PPE) are vital. A survey conducted by Husain MA et al. (2021) in Saudi Arabia indicated that the degree of knowledge about COVID-19 preventive measures among physiotherapists is considered high, but few lacked knowledge of the application of PPE [12]. Physiotherapists must be knowledgeable about the disease symptoms, mode of transmission, risk mitigation strategies, diagnostic measures, and practice guidelines for COVID-19.

In March 2020, Thomas et al. provided physiotherapy recommendations that were internationally endorsed by several associations worldwide. This includes planning and preparing a physiotherapy workforce, a screening tool for determining the category of patients requiring physiotherapy, recommendations on selecting physiotherapy treatments, and using PPE, which plays an essential role in preventing the spread of the virus. An update to these recommendations has been published in 2022 [13, 14].

An evidence-based national consensus was conducted in 2020 to provide specific physiotherapy practice recommendations in the COVID-19 acute care Indian setup, which was endorsed by the Indian Association of Physiotherapy (IAP). The aim of the treatment and recommendation were categorized according to the patient's presentation, i.e., mechanically ventilated or nonmechanically ventilated in the COVID-19 ICU [11, 15]. Both guidelines are similar, except that the international guideline states that there is no evidence for using incentive spirometry in COVID-19 patients. In contrast, the nationally accepted guideline advises its use in stable patients to improve volume and ventilation.

Chest physiotherapy is most effective in reducing the pulmonary infection rate, and it can improve quality of life by improving respiratory function. It includes percussion, vibration, postural drainage, and airway suctioning techniques. Chest physiotherapy should be given only when beneficial and individualized according to the patient's presentation [9, 16]. It has been found that active breathing exercises have improved pulmonary ventilation, mobilization, and excretion of secretions and stimulate respiratory muscles. Positioning a patient posttreatment or using positional treatment alone helps in increasing the ventilation-perfusion ratio. Exercise therapy improves immunity and reduces complications favoring functional recovery in patients affected by COVID-19 [17].

Due to the increase in COVID-19 patients admitted to ICUs, the pandemic has challenged physiotherapists to apply their knowledge and skills to treat these patients [17]. Since this is an evolving area of practice and even though physiotherapy interventions have been proven beneficial, the knowledge, attitudes, and practices among physiotherapists in Indian ICUs remain unexplored. Thus, the study aims to explore physiotherapists' knowledge, attitude, and practice patterns in COVID-19 ICUs in India.

## 2. Methods and Methodology

### 2.1. Procedure

It was a cross-sectional survey conducted from February 2022 until January 2023.

The study was approved by the Institutional Scientific and Ethical Committee (protocol no. IEC KMC MLR 01/2022/37), Kasturba Medical College, Manipal Academy of Higher Education, Mangalore.

Physiotherapists of either gender, with a minimum experience of 1 month, or who have worked in the COVID-19 ICUs of India could participate.

The survey questionnaire was prepared using Google Forms. It consisted of 23 questions divided into three sections and the demographics of the respondents like age, gender, the highest level of qualification, the state where they work, and months of experience. The investigators designed the questionnaire to focus on the knowledge, attitude, and practice of physiotherapists working in COVID-19 ICUs in India.

Section A included seven questions taken from the guidelines aimed to assess physiotherapists' knowledge regarding physiotherapy guidelines, Section B included four questions regarding physiotherapists' attitudes toward patient care, and Section C had twelve questions focused on assessing their practice patterns in COVID-19 ICUs. Mostly, the items were close-ended/multiple-choice questions to avoid self-bias. An extra column was added where the respondent could add to the already provided options where relevant. In some places, choices of multiple answer selections were also offered. The entire questionnaire (given in Appendix 1) took about 15 min to fill out.

Hospitals having COVID-19 ICUs were shortlisted by the National Accreditation Board for Hospitals (NABH), the Medical Council of India (MCI), and IAP websites, and 600 questionnaires were mailed requesting to circulate among physiotherapists. The questionnaires were also distributed through e-mail, Facebook Messenger, or WhatsApp. They included a hyperlink that directed participants to the survey on a web page. The investigators priorly informed the scope and objective of the study. Participants filled out the questionnaires only after giving their consent for the study.

To ensure a good response rate, a span of 3 weeks was given to participants to return the filled questionnaire. If a response was not obtained within the stipulated period, then two reminders were sent with a gap of one month. After that, nonresponders were excluded from the study.

Before starting the study, the survey questionnaire was submitted to 5 experts: cardiopulmonary physiotherapists (*n* = 4) and a faculty from the Department of Community Medicine (*n* = 1). All feedback was taken, and appropriate changes were made. Later, the content validity index (CVI) was used to measure the validity of the questionnaire. This is the most widely used approach for the development of the content validity of an instrument. The questionnaire's relevance was calculated similarly on a 5-point scale: 1: irrelevant, 2: slightly relevant, 3: somewhat relevant, 4: moderately relevant, and 5: extremely relevant through Google Forms.

To judge a scale as having excellent content validity, it should be composed of items with I-CVIs that meet Lynn's (1986) criteria (I-CVI of 1.00 with 3–5 experts and a minimum I-CVI of 0.78 for 6–10 experts) and it should have an S-CVI/Ave of 0.90 or higher [18].

The final draft of the survey was designed according to the suggestions given by the subject experts.

### 2.2. Data Analysis

After the data were collected, they were exported to Jamovi (version 2.3.21) software for analysis.

Frequency, cumulative frequency, and percentages of all responses have been obtained along with bar charts to summarize the categorical data considered in the study.

Furthermore, we used one-way ANOVA to determine whether there is statistical evidence of a significant association between different variables.

## 3. Result

### 3.1. Content Validation

The validity of the questionnaire was determined using CVI and is explained in Table 1.

The proportional relevance (PR) for expert 1 is 0.9, expert 2 is 0.9, expert 3 is 1, expert 4 is 0.9, and expert 5 is 1. The score content validity average based on I-CVI is 0.97, the score content validity average based on PR is 0.94, and the score content validity average based on universal agreement is 0.91.

From the values obtained, we can conclude that S-CVI Ave based on I-CVI, S-CVI Ave based on PR, and S-CVI Ave based on UA meet a satisfactory level. Thus, the scale of the questionnaire has achieved satisfactory content validity [18].

### 3.2. Response

**Figure 1:**
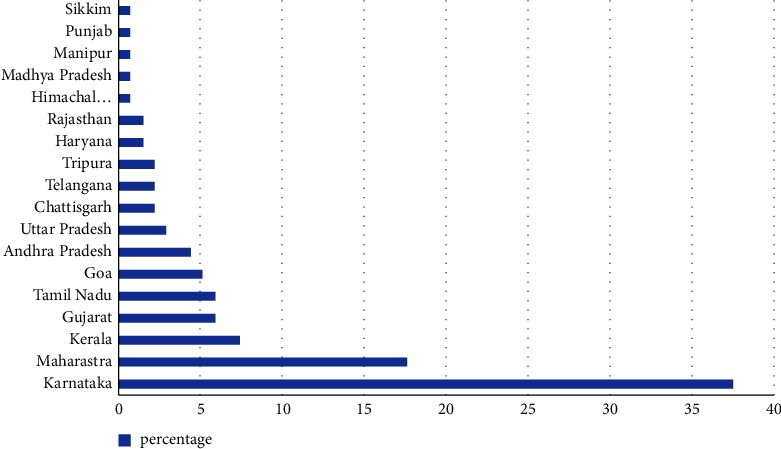
Response rate obtained from different states.

A total of 600 questionnaires were emailed to physiotherapists across India, with a total of 136 completed and returned. This made for a response rate of 22.6%. The responses were received from 18 states, including Karnataka, Maharashtra, Kerala, Gujarat, Tamil Nadu, Goa, Andhra Pradesh, Uttar Pradesh, Chhattisgarh, Telangana, Tripura, Haryana, Rajasthan, Himachal Pradesh, Madhya Pradesh, Manipur, Punjab, and Sikkim. The majority of the responders were from Karnataka (*n* = 51 (37.5%)), Maharashtra (*n* = 24 (17.6%)), and Kerala (*n* = 10 (7.4%)) (Figure 1).

### 3.3. Demographic Characteristics and Qualifications of the Participants

The details about the demographic characteristics and qualifications are given in Table 2. Of 136 participants, 89 were female (65.4%) and 47 were male (34.6%). The highest level of qualification was BPT (*n* = 69 (50.7%)), followed by MPT (*n* = 62 (45.6%)) and Ph.D. (3 (3.7%)). A total of 61 were designated physiotherapists (44.9%), 36 were postgraduates (26.5%), 16 were interns (11.8%), 10 were assistant professors (7.4%), 9 were consultant physiotherapists (6.6%), 2 were lecturers (1.5%), and 2 were associate professors (1.5%). Twenty-seven had less than a month of experience, 52 had 1–3-month experience, 23 had 3–5-month experience, 13 had 6–12-month experience, and 21 had more than a year of experience working in the COVID ICU.

### 3.4. Knowledge

**Figure 2:**
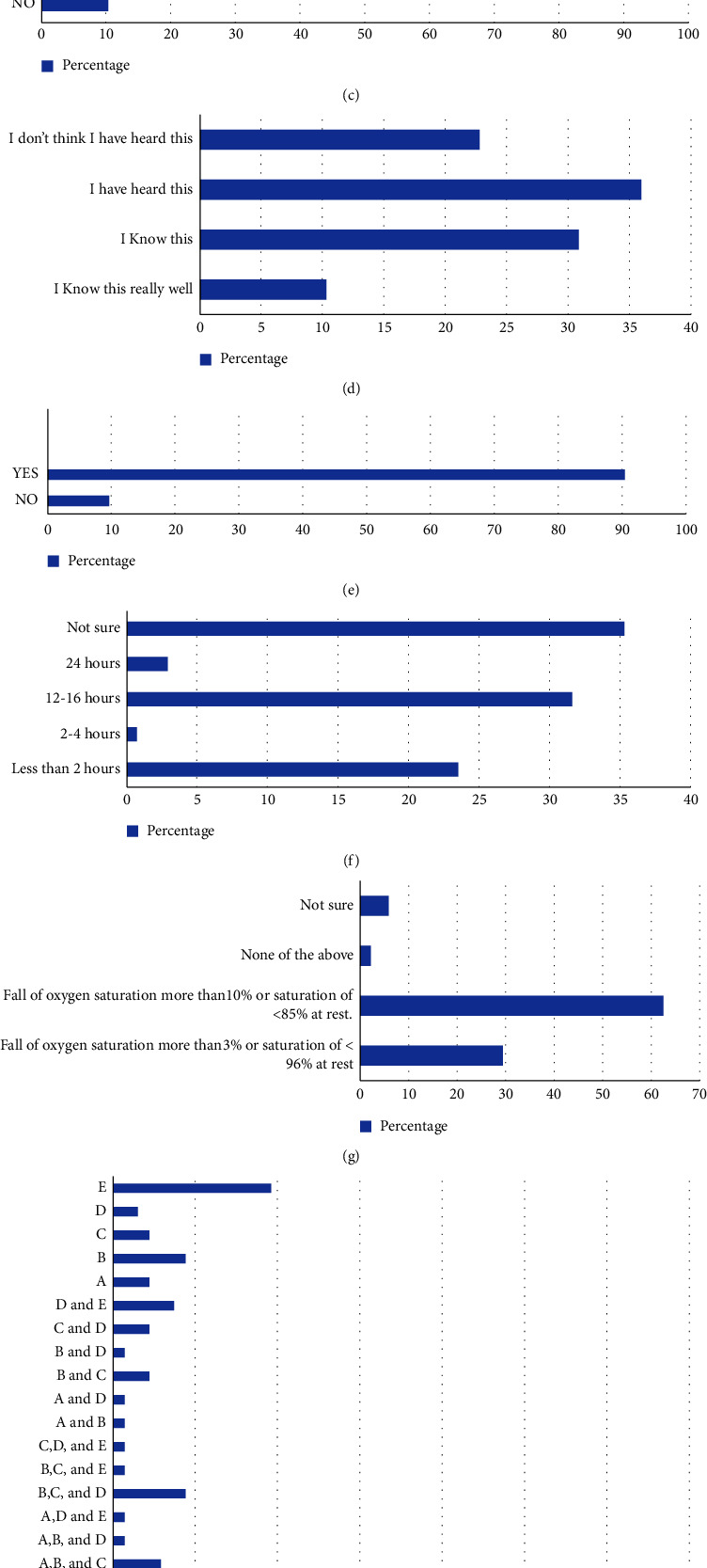
(a) Response rate for “did the physiotherapists, including you, receive any special training before you started working in COVID-19 ICU?” (b) Response rate for “if yes, what type of training?” (c) Response rate for “are you aware of the technique and sequence of donning and doffing PPE?” (d) Response rate for “are you familiar with COVID-19 awake repositioning proning (CARP) protocol?” (e) Response rate for “do you think prone positioning for patients with severe ARDS is applicable in COVID patients too?” (f) Response rate for “if yes, then what is the recommended duration per day?” (g) Criteria for closed monitoring during treatment. (h) Response rate for “which of the following categories of patients are indicated for physiotherapy?” (A) Mild symptoms such as dry cough and fever; without significant respiratory compromise, low-level oxygen requirement (e.g., oxygen flow ≤5 L/min for SpO_2_ ≥90%), the patient was able to cough effectively. (B) Mild symptoms and/or pneumonia and coexisting respiratory or neuromuscular comorbidity (e.g., cystic fibrosis, neuromuscular comorbidity, and chronic obstructive pulmonary disease) and current or anticipated difficulties with secretion clearance. (C) Mild symptoms and/or pneumonia and evidence of exudative consolidation with difficulty clearing or inability to clear secretion inadequately. (D) Severe symptoms suggesting pneumonia/ lower respiratory tract infection, e.g., increasing oxygen requirements, fever, difficulty breathing, frequent/severe/productive cough, and chest X-ray/ CT scan/ lung ultrasound showing changes consistent with consolidation. (E) Any patient at significant risk of developing or with mild evidence of significant functional limitation, e.g., multiple comorbidities, a patient at risk of ICU-acquired weakness. (i) Response rate for “do you think that strengthening skeletal muscles and recovery of ADL should be contraindicated in patients admitted in COVID ICUs, as they can increase the load on the respiratory system and increase the risk of distress?”

Of the total respondents, 89 physiotherapists had not received any special training before being deployed in COVID-19 ICUs and 47 received special training, including donning and doffing of PPE (*n* = 18 (13.2)), ICU management (*n* = 3 (2.2%)), precautions (*n* = 8 (5.9%)), PR (*n* = 3 (2.2%)), ICU management and precautions (*n* = 7 (5.1%)), and donning and doffing of PPE and other precautions (*n* = 2 (1.5%)) (Figures 2(a) and 2(b)).

A total of 122 (89.7%) were aware, and 14 (10.3%) were unaware of the PPE donning and doffing sequence (Figure 2(c)).

Only 14 physiotherapists knew the COVID awake repositioning proning (CARP) protocol really well (10.3%), 42 knew the protocol (30.9%), 49 had heard of it (36%), and 31 did not think they had heard of it (22.8%) (Figure 2(d)). While the majority of the responders (*n* = 123 (90.4%)) thought prone positioning was applicable in COVID patients, only (*n* = 43 (31.6%)) were aware of the recommended duration for the CARP protocol (Figures 2(e) and 2(f)). Criteria for close monitoring “fall of oxygen saturation more than 3% or saturation of <96% at rest” were chosen by 40 (29.4%) responders, while 85 (62.5%) chose “fall of oxygen saturation more than 10% or saturation of <85% at rest, 8 (5.9%) were not sure, and 3 chose “none of the above” (Figure 2(g)). Only 39 (28.7) responders were able to choose the category of patients rightly indicated for physiotherapy, i.e., “mild symptoms and/or pneumonia and coexisting respiratory or neuromuscular comorbidities and current or anticipated difficulties with secretion clearance; mild symptoms and/or pneumonia and evidence of exudative consolidation with difficulty clearing or inability to clear secretion inadequately; severe symptoms suggesting pneumonia/lower respiratory tract infection; chest X-ray/CT scan/lung ultrasound showing changes consistent with consolidation; and any patient at significant risk of developing or with mild evidence of significant functional limitation” (Figure 2(h)).

A total of 52 (38.2%) rightly thought that strengthening skeletal muscles and recovery of activities of daily living should be contraindicated in patients admitted to COVID-19 ICUs, as they can increase the load on the respiratory system and increase the risk of distress, while 64 (47.1%) think this is not contraindicated. The rest 20 (14.7%) are still determining (Figure 2(i)).

In total, only 45.47% of the participants gave the appropriate answers to knowledge-related questions.

### 3.5. Attitude

**Figure 3:**
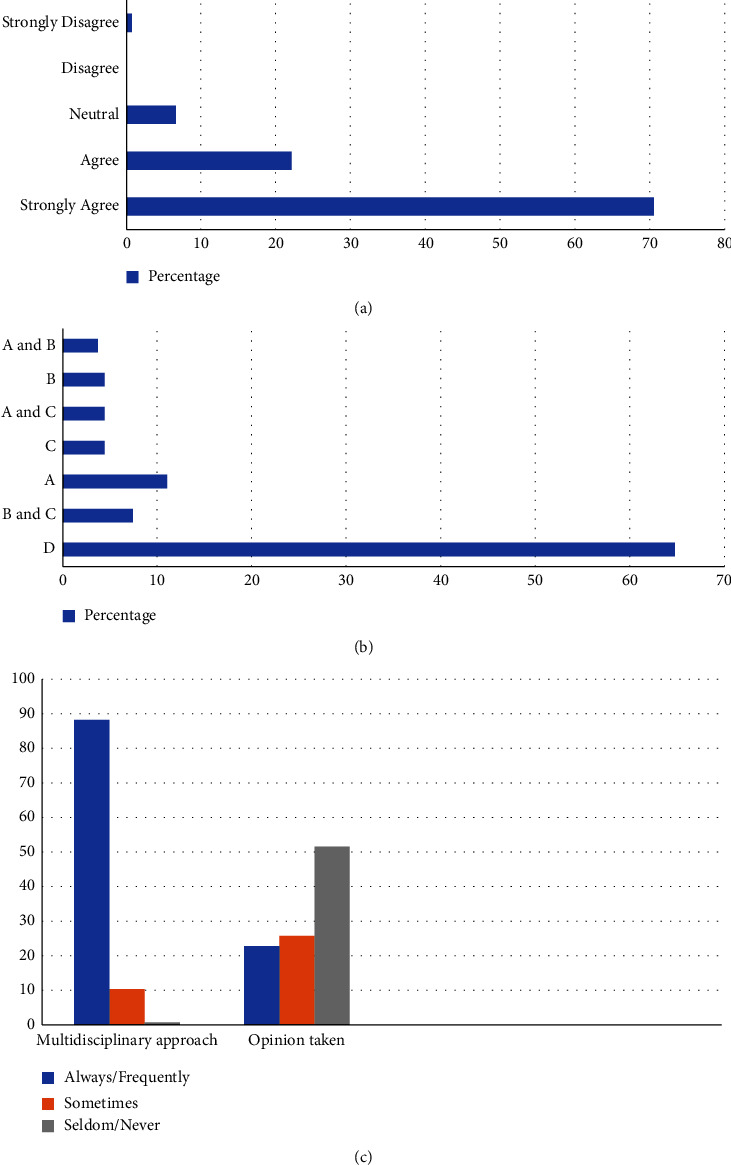
(a) Response rate for “please assume for a moment about exposure to the therapist and answer the question, physiotherapy service is essential in COVID-19 ICU?” (b) Response rate for “which phase of COVID do you think physiotherapy should be initiated?” (A) Acute/mild. (B) Critical. (C) Postacute and discharge. (D) All of the above. (c) Response rate for “do you follow a multidisciplinary approach?” and “is the physiotherapists' opinion taken before shifting the patient out of COVID ICU?”

When asked if physiotherapy is essential in COVID-19 ICUs despite the risk of exposure, 96 (70.6%) strongly agreed 30 (22.1%) agreed, 9 (6.6%) were neutral, and 1 (0.7%) strongly disagreed (Figure 3(a)).

A total of 88 (64.7%) thought physiotherapy should be initiated during all phases of COVID, 10 (7.4%) during critical and postdischarge, 15 (11%) during acute/mild, 6 (4.4%) during postacute and discharge, 6 (4.4%) during acute/mild and postacute discharge, 6 (4.4%) during critical, and 5 (3.7%) during acute/mild and critical (Figure 3(b)).

A multidisciplinary approach was “always or frequently” by 121 (88.9%), followed by “sometimes” by 14 (10.3%) and “seldom or never” by 1 (0.7%). When asked if a physiotherapist's opinion was taken before shifting the patient out of COVID-19 ICUs, 31 (22.8%) chose “always or frequently,” 35 (25.7%) chose “sometimes,” and 70 (51.5%) chose “seldom or never” (Figure 3(c)).

### 3.6. Practice

**Figure 4:**
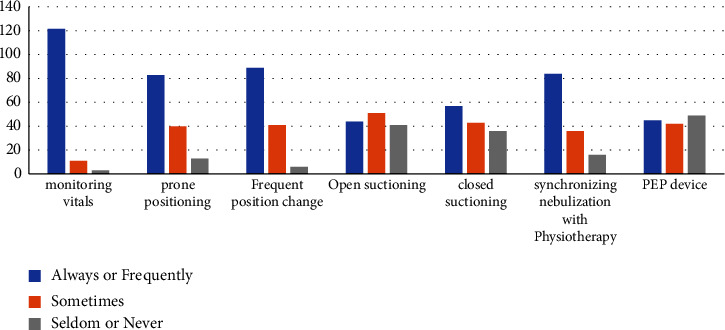
Different physiotherapy practices.

The frequencies of different physiotherapy practices are given in Figure 4.

The majority of the physiotherapists (122 (89.7%)) had “always or frequently” opportunities to monitor and record vital details pretreatment and posttreatment, 11 (8.1%) “sometimes,” and 3 (2.2%) “seldom or never.” The prone position was “always or frequently” used by 83 (61%), “sometimes” by 40 (29.4%), and “seldom or never” by 13 (9.5%).

A total of 89 (65.4%) could “always or frequently” recommend a change in patient position, 41 (30.1%) “sometimes,” and 6 (4.4%) “seldom or never” had this opportunity. 51 (37.5%) “sometimes” used open suction in COVID-19 ICUs, 44 (32.3%) used it “always or frequently,” and 41 (30.2%) were “seldom or never” involved.

Closed suctioning was used “always or frequently” by 57 (41.9%), “sometimes” by 43 (31.6%), and “seldom or never” by 36 (26.5%). 84 (61.8%) could “always or frequently” synchronize nebulization with bronchial hygiene therapy, 36 (26.5%) could “sometimes,” and 16 (11.8%) “seldom or never” had this opportunity.

When asked about using PEP devices, 45 (33.1%) chose “always or frequently,” 42 (30.9%) chose “sometimes,” and 49 (36%) “seldom or never” used. The commonly used PEP devices were Acapella (22 (16.2%)), Flutter (12 (8.8%)), or both (18 (13.2%)). Other devices, such as Aerobika (1 (0.7%)) and RC-Cornet (1 (0.7%)), were also used.

A total of 64 (47%) “always or frequently” provided early mobilization to mechanically ventilated patients, 47 (34.6%) sometimes,” and 25 (18.4%) “seldom or never” preferred it.

While 86 (63.3%) “always or frequently” and 27 (19.9%) “sometimes” had the opportunity to ambulate stable patients within the ICU, 23 (16.9%) “seldom or never” had.

Four case-based questions were asked, and the responses are given in Tables 3–6.

For patients presenting with saturation, mild or no change at rest (SpO_2_ of 92–94%); symptoms: none; HD CT score: <8; oxygen support: low-flow oxygen or room air/home isolation; more than 80% of the responders marked “always or frequently” using the following methods: breathing exercise (133 (97.8%)), relaxation exercise (119 (87.5%)), thoracic expansion exercise (127 (93.4%)), ACBT-FET (108 (79.4%)), and active range of motion (114 (83.8%)). Other always or frequently used techniques were prone positioning (59 (43.4%)), postural drainage (83 (61%)), incentive spirometry (95 (69.8%)), and ambulation (95 (69.9%)). Percussions (62 (45.6%)), vibration (57 (41.9%)), and Acapella (48 (35.3%)) were marked “sometimes,” and “seldom or never” used techniques were mechanical insufflation-exsufflation (74 (54.4%)), closed suctioning (75 (55.2%)), open suctioning (72 (52.9%)), and IPPB (80 (58.8%)).

Patients with HD CT scan score of 9–19, presenting with symptoms of fever, cough, breathlessness, and saturation drop during activity, requiring high flow oxygen (HFO) system/venturi support; more than 70% of the responders marked “Always or frequently” using the following methods: breathing exercise (114 (83.8%)), relaxation exercise (116 (85.3%)), thoracic expansion exercise (105 (77.2%)), postural drainage (96 ((70.6%)), and ACBT- FET (101 (74.3%)). Other always or frequently used techniques were CARP protocol (60 (44.2%)), prone positioning (65 (47.8%)), incentive spirometry (78 (57.4%)), percussion (63 (46.3%)), vibration (66 (48.8%)), active range of motion (89 (65.5%)), and active-assisted range of motion (85 (62.5%)).

Acapella (48 (35.3%)), Flutter (49 (36%)), and abulation (59 (43.4%)) were marked “sometimes,” and seldom or never used techniques were mechanical insufflation-exsufflation (70 (51.5%)), closed suctioning (43 (31.7%)), IPPB (71 (52.2%)), and balance training (57 (41.9%)).

Patients with HD CT scan score of >15, presenting with symptoms of altered mental status, signs of ARDS, and requiring invasive or noninvasive ventilator with high FiO_2_ and PEEP to maintain oxygenation”; more than 50% of the responders marked “Always or frequently” using the following methods: relaxation exercise (74 (54.4%)), prone positioning (80 (58.8%)), postural drainage (99 (72.8%)), percussion (88 (64.8%)), vibration (84 (1.7%)), closed suctioning (86 (63.3%)), passive range of motion 85 (62.5%), and active assisted range of motion 77 (56.7%). Other always or frequently used techniques were breathing exercise (65 (47.7%)), thoracic expansion exercise (53 (38.9%)), and CARP protocol (62 (45.6%)). Mechanical insufflation-exsufflation (50 (36.8%)), open suctioning (58 (42.7%)), and active range of motion (65 (47.8%)) were used sometimes. Seldom or never used techniques were incentive spirometry (59 (43.4%)), IPPB (67 (49.3%)), Acapella (75 (55.2%)), Flutter (75 (55.2%)), balance training (81 (59.6%)), and ambulation (73 (53.7%)).

Patients presenting with symptoms ARDS, multiorgan failure, sepsis, shock, and requiring mechanical ventilator or extra corporeal membrane oxygenation (ECMO) with high FiO_2_ and PEEP to maintain oxygenation”; more than 50% of the responders marked “Always or frequently” using the following methods: prone positioning 71 (52.1%), postural drainage 88 (64.6%), percussion 87 (63.9%), vibration 84 (61.7%), closed suctioning 51 (58%), and passive range of motion 76 (55.8%). Other always or frequently used techniques were relaxation exercise (61 (44.9%)), CARP protocol (51 (37.5%)), mechanical insufflation-exsufflation (48 (35.3%)), open suctioning (57 (42%)), and active-assisted range of motion (66 (48.6%)). Active range of motion (53 (38.9%)) was used occasionally, and seldom or never used techniques included breathing exercise (56 (41.2%)), thoracic expansion exercise (61 (44.9%)), incentive spirometry (64 (47%)), ACBT-FET (68 (50%)), IPPB (66 (48.5%)), Acapella (72 (52.9%)), Flutter (72 (52.9%)), balance training (85 (62.4%)), and ambulation (80 (58.8%)).

Table 7 shows the association of different characteristics with knowledge, attitude, and practice.

Subgroup analysis revealed that there is a significant association for knowledge with qualification, designation, age, and duration of work. There is also a significant association for practice with qualification, age, and duration of work.

## 4. Discussion

The present study has assessed the knowledge, attitude, and practice of physiotherapists working in COVID ICUs in India.

COVID-19 breakout was the most critical health emergency in the recent decade; such an event with exceptional magnitude was required to increase ICU capacity, which in turn demanded appropriately trained healthcare staff as the process included the deployment of non-ICU healthcare professionals [19, 20].

Several studies were conducted to assess the knowledge and attitude of physiotherapists about COVID-19; these were conducted regarding the source of awareness, clinical symptoms, diagnostic measures, mode of transmission, risk mitigation, and preventive measures. These studies found that physiotherapists are sufficiently knowledgeable about COVID-19 [21–24]. However, these studies had not assessed (i) the knowledge and attitude of physiotherapists, specifically in those who worked in COVID-19 ICUs, and (ii) awareness of gold standard rehabilitation protocols. Thus, we aimed to assess physiotherapists' knowledge of the existing physiotherapy guidelines for COVID-19. To the best of our knowledge, this is the first study to assess the knowledge of physiotherapists regarding the existing physiotherapy COVID-19 guidelines. The findings of our study revealed that only 34.6% of the physiotherapists received special training, including PPE donning and doffing technique, management of patients in the ICU, COVID-19 precautions, and pulmonary rehabilitation, before being deployed in the COVID-19 ICU. Healthcare providers working in COVID require an introduction to the diagnosis and newer anticipated needs such as proning and positioning, maintaining vascular catheters and dialysis circuits, sedation and administering vasoactive medication, continuous positive airway pressure services, and particularly on PPE and infection control techniques [25]. Physiotherapists being an essential part of the ICU team are required to have skills in managing the needs of patients according to the symptoms, which include awareness of presuction positioning, suctioning techniques, ability to judge and increase FiO_2_, and use of maneuvers such as percussion, vibrations, and shaking [26]. Although many did not receive any training, most of them knew the PPE donning and doffing sequence (89.7%); this is in line with other studies assessing physiotherapists' knowledge about COVID-19 infection prevention control measures [12, 27]. 41.2% were aware of the CARP protocol, but only 31.6% knew the recommended duration. According to the guidelines, close monitoring is indicated when there is a fall in oxygen saturation of more than 3% or saturation below 96%, of which 71.7% of the responders were unaware. In our study, it was found that only 28.7% of responders were able to choose the category of patients indicated for physiotherapy intervention correctly, and this is not in accordance with a recent study conducted that stated 90% of the participants were aware of physiotherapy-specific guidelines for treating patients with COVID-19 [28]. In total, only 45.47% of the participants gave the appropriate answers to knowledge-related questions, indicating insufficient knowledge about the existing guidelines.

The exploration of the attitude of physiotherapists toward working in ICUs revealed that 70.6% of the responders thought that physiotherapy was a vital component of ICU management despite the risk of exposure, and this was in line with the findings obtained in a study by Mohamed et al., in Saudi Arabia, about knowledge, attitude, practice, and perceived job stress among physiotherapists, which revealed that 75.8% of the responders expressed their disagreement that they are concerned about getting COVID-19 while participating in inpatient care [29]. This shows a positive attitude of physiotherapists consistently toward COVID-19, despite self-exposure risk, by realizing their crucial role in their management. Moreover, most of the responders in our study thought that physiotherapy as a multidisciplinary approach should be initiated during all phases of COVID-19, be it in the ICU, where the patient is critically ill, or even after discharge. It was estimated that 80% of the infected patients with COVID-19 developed one or more long-term symptoms, lasting from several months to a year or more. Fatigue, headache, attention disorder, hair loss, dry cough, productive cough, and dyspnea were some of the most common symptoms. Research suggests that multidisciplinary teams are crucial to developing preventive measures, rehabilitation techniques, and clinical management strategies with whole-patient perspectives designed to address long-term COVID-19 care [30]. While the role of the physiotherapist in the COVID ICU is paramount, 51.5% of the responders in our study chose “seldom/never” when asked if their opinion was taken before deciding to shift the patient to the ward. The findings of our study indicate that physiotherapists working in COVID-19 ICUs showed a positive attitude toward treating patients with COVID-19.

The present study showed that the majority of the physiotherapists consistently monitored vitals pretreatment and posttreatment; this is in accordance with the physiotherapy guidelines for patients with COVID-19, which states that all must be screened, assessed, and monitored continuously during and postphysiotherapy treatment [14, 15]. Prone positioning is highly recommended in critically ill patients infected with COVID-19. It was found to improve the oxygen level by optimizing lung recruitment and improving the ventilation/perfusion index. However, this is not indicated in all patients. Several contraindications, such as increased intracranial pressure, spinal instability, pregnancy, and open abdominal wounds, should be performed only when sufficient technical resources are available [17]. The guidelines recommended a prone position for 12–16 hours a day. Our responders commonly practice prone and frequent changes in position. Similar findings have been found in an earlier study conducted in Brazil [31]. In the current study, 41.9% of the physiotherapists were always involved in closed suctioning, while 32.3% still always used open suctioning despite the risk of creating an airborne transmission of COVID-19 treatment. Our finding contradicts Trojman A et al., whose results stated that 97.8% of physiotherapists adhered to the guidelines and did not use open suctioning in ventilated patients.

Patients admitted to the acute care setup can be categorized into mild, moderate, and severe. Recommendations for physiotherapy intervention in these categories have been provided in the guidelines; it is the responsibility of physiotherapists to use these recommendations along with their clinical judgment. Our study participants showed that they follow best practice behavior in treating patients in COVID-19 ICUs, which aligns with physiotherapy guidelines for COVID-19. In patients presenting with mild symptoms, i.e., patients on low-flow oxygen, room air, or home isolation, breathing exercise was used by 97.8% of them. The most frequently used airway clearance techniques were ACBT, ACBT-FET (79.4%), and postural drainage (61%). Incentive spirometry was used by 69.8% of the participants, which can improve lung volume and ventilation in patients on nasal prongs or room air. Early mobilization is highly recommended in these patients as this could prevent secondary complications such as ICU-acquired weakness; in the present study, active range of motion exercises were the choice of mobilization therapy, and a good number of participants (69.9%) were always involved in ambulating the patients within the ICU.

In patients presenting with moderate symptoms, i.e., patients receiving a high-flow oxygen (HFO) system or Venturi mask and whose SpO_2_ drops during activity, the respiratory physiotherapy techniques most commonly used by our participants were relaxation, active breathing, and bronchial hygiene therapy, including thoracic expansion exercises, postural drainage, percussion, and vibration. More than half of the responders practice therapeutic positioning, including CARP and prone positioning, which is advised in these patients to increase oxygen saturation and delay or reduce the need for intubation. The different mobilization therapies used range from active, active-assisted, and passive range of motion depending on the patient's level of consciousness and ability to follow commands, and only 29.4% of participants were involved in ambulating these patients. This could be because of several reasons such as fatigue, breathlessness, and desaturation with activity; thus, close monitoring to screen for activity-induced desaturation is mandatory, and when required, oxygen titration in consultation with a physician should be performed [14, 15].

The physiotherapy recommendations given for patients on MV and ECMO are similar. The result from our study also shows similar practice patterns in both categories. The most frequently used techniques were prone positioning, postural drainage, and closed suctioning. In mobilization therapy, passive- and active-assisted ranges of motion are used. Although ambulation is not contraindicated in mechanically ventilated patients or those on ECMO, most of our responders seldom or never practiced it. This could be due to a lack of personnel or needing to be experienced in doing so.

Subgroup analysis showed a significant association for knowledge and practice with qualification, designation, age, and duration of work. This could be due to greater exposure to similar situations, patient interactions, etc. Those specialised in cardiopulmonary physiotherapy would have better knowledge about COVID-19 which in turn will increase with other factors such as experience, exposure, or even age [32].

However, there is no association of attitude with any characteristics, and all expressed positive attitudes. A good attitude is vital for a healthcare professional. There is a big discussion going on all over the world as to how to improve the attitude of health professionals. In recent times, a new curriculum called Attitudes, Ethics, and Communication (AETCOM) has been integrated for undergraduate students which will run across years. The learning outcomes enable them to assess the communication needs of the patient/community. As India has made AETCOM compulsory in the first year, core skills in communication (listening, interpreting, body language, writing, reading, and reasoning) are inculcated, and the learner can communicate effectively with patients, the community, and colleagues in a respectful nonthreatening, nonjudgmental, and empathetic manner [33].

This study has some limitations. A smaller sample size and bias of responders toward the states of Karnataka, Maharashtra, and Kerala could mean that the study results may not reflect the practice patterns of physiotherapists in Indian COVID-19 ICUs. More open-ended questions would have been able to provide better qualitative data.

## 5. Conclusion

The results of the current study may add to the limited evidence available regarding the knowledge of physiotherapists about the existing COVID-19 practice guidelines, their attitude toward the disease, and the practice pattern used in COVID-19 ICUs. The results show that the number of females working in COVID ICUs was greater than that of men, and their highest level of qualification was BPT followed by MPT. The knowledge and practice patterns were better among physiotherapists with higher qualifications and years of experience. The study suggests that the attitude of physiotherapists is appropriate and there is no significant association between qualification, experience, or designation, which shows that all the physiotherapists irrespective of the differences show promising attitudes toward disease and their patients, while knowledge regarding the physiotherapy guidelines for COVID-19 needs to be more satisfactory. This could be because of the need for more educational support for them. Most physiotherapists did not obtain any training before being deployed in COVID-19 ICUs. Additional education and organizational support may enhance the quality of service provided to patients and prepare the physiotherapists to make appropriate decisions, which may also reduce their work-related stress.

### 5.1. Clinical Significance

The study results will help us understand physiotherapists' knowledge regarding the existing COVID-19 guidelines, their attitude toward the disease and patient management, and practices in COVID-19 ICUs. The study results found that most physiotherapists involved in COVID-19 patient care had bachelor's degrees and lacked training; there is also a significant association of knowledge and practice with qualification, age, and experience. Thus, the study shed light on the need to impart specific educational training or awareness programs before deputing a physical therapist, especially if they are new graduates with no prior experience in a similar field for intensive care in COVID-19 patients. This would help improve the physiotherapy service provided, thus improving the treatment outcome which is the ultimate goal.

### 5.2. Future Recommendations

Future studies need to include a qualitative approach to obtain more details about the challenges and components of physiotherapy practice.

## Figures and Tables

**Table 1:** Response of each question from 5 experts, along with expert agreement, I-CVI, PR, and UA, where I-CVI is item content validity, UA is universal agreement, PR is proportion relevance, S-CVI Ave is score content validity average based on I-CVI, S-CVI Ave is score content validity average based on PR, and S-CVI Ave is score content validity average based on UA.

Sections	Question	Expert 1	Expert 2	Expert 3	Expert 4	Expert 5	Expert in agreement	I-CVI	UA
A	Q1	5	4	5	5	5	5	1	1
	Q2	5	4	5	4	5	5	1	1
	Q3	5	4	5	4	5	5	1	1
	Q4	4	4	5	3	5	4	0.8	0
	Q5	4	4	5	5	5	5	1	1
	Q6	5	4	5	5	5	5	1	1
	Q7	5	4	5	5	5	5	1	1

B	Q8	5	5	5	5	5	5	1	1
	Q9	4	5	5	5	5	5	1	1
	Q10	5	4	4	5	5	5	1	1
	Q11	2	3	4	5	5	3	0.6	0

C	Q12	5	4	5	5	5	5	1	1
	Q13	5	5	5	5	5	5	1	1
	Q14	5	5	5	5	5	5	1	1
	Q15	5	5	5	5	5	5	1	1
	Q16	5	5	5	5	5	5	1	1
	Q17	5	4	5	5	5	5	1	1
	Q18	5	5	5	4	5	5	1	1
	Q19	4	5	5	4	5	5	1	1
	Q20	4	5	5	5	5	5	1	1
	Q21	4	5	5	5	5	5	1	1
	Q22	4	5	5	5	5	5	1	1
	Q23	4	5	5	5	5	5	1	1

	PR	0.9	0.9	1	0.9	1		S-CVI Ave = 0.97	S-CVI Ave = 0.91

S-CVI average based on PR = 0.94.

**Table 2:** Demographics and qualifications of the participants.

Characteristics	*n*	%
Gender		
Female	89	65.4
Male	47	34.6
Qualification		
BPT	69	50.7
MPT	62	45.6
Ph.D.	3	3.7
Designation		
Physiotherapist	61	44.9
Postgraduates	36	26.5
Interns	16	11.8
Assistant professors	10	7.4
Consultant physiotherapists	9	6.6
Lecturers	2	1.5
Experience in COVID-19 ICU		
Less than a month	27	19.9
1–3-month	52	38.2
3–5-month	23	16.9
6–12-month	13	9.6
More than a year	21	15.4

**Table 3:** Frequency of different treatment methods used in patients presenting with saturation: mild or no change at rest (SpO_2_ of 92–94%); symptoms: none; HD CT score: <8; oxygen support: low-flow oxygen or room air/home isolation.

	Always/frequently	Sometimes	Seldom/never
Breathing exercise	133 (97.8%)	3 (2.2%)	
Relaxation exercise	119 (87.5%)	16 (11.8%)	1 (0.7%)
Thoracic expansion exercise	127 (93.4%)	7 (5.1%)	2 (1.5%)
CARP protocol	51 (37.5%)	31 (22.8%)	54 (39.7%)
Prone positioning	59 (43.4%)	52 (38.2%)	25 (18.4%)
Postural drainage	83 (61%)	40 (29.4%)	13 (9.6%)
Incentive spirometry	95 (69.8%)	25 (18.4%)	16 (11.7%)
ACBT-FET	108 (79.4%)	21 (15.4%)	7 (5.1%)
Mechanical insufflation-exsufflation	29 (21.3%)	33 (24.3%)	74 (54.4%)
Percussion	52 (38.2%)	62 (45.6%)	22 (16.2%)
Vibration	57 (41.9%)	56 (41.2%)	23 (17%)
Closed suctioning	37 (27.2%)	24 (17.6%)	75 (55.2%)
Open suctioning	35 (25.8%)	29 (21.3%)	72 (52.9%)
IPPB	28 (20.6%)	28 (20.6%)	80 (58.8%)
Acapella	44 (32.3%)	48 (35.3%)	44 (32.3%)
Flutter	47 (34.6%)	41 (30.1%)	48 (35.3%)
Passive range of motion	57 (41.9%)	49 (36%)	30 (22.1%)
Active-assisted range of motion	89 (65.4%)	29 (21.3%)	18 (13.2%)
Active range of motion	114 (83.8%)	19 (14%)	3 (2.2%)
Balance training	55 (40.5%)	47 (34.6%)	34 (25%)
Ambulation	95 (69.9%)	33 (24.3%)	8 (5.9%)

**Table 4:** Frequency of different treatment methods used in patients presenting with saturation: drop in SpO_2_ during activity; symptoms: fever, cough, and breathlessness; HD CT scan: 9–19; oxygen support: high-flow oxygen (HFO) system/Venturi masks.

	Always/frequently	Sometimes	Seldom/never
Breathing exercise	114 (83.8%)	14 (10.2%)	8 (5.9%)
Relaxation exercise	116 (85.3%)	15 (11%)	5 (3.7%)
Thoracic expansion exercise	105 (77.2%)	18 (13.2%)	13 (9.6%)
CARP protocol	60 (44.2%)	26 (19.1%)	50 (36.8%)
Prone positioning	65 (47.8%)	54 (39.7%)	17 (12.5%)
Postural drainage	96 (70.6%)	32 (23.5%)	8 (5.9%)
Incentive spirometry	78 (57.4%)	32 (23.5%)	26 (19.2%)
ACBT-FET	101 (74.3%)	21 (15.4%)	14 (10.3%)
Mechanical insufflation-exsufflation	38 (27.9%)	28 (20.6%)	70 (51.5%)
Percussion	63 (46.3%)	53 (39%)	20 (14.7%)
Vibration	66 (48.8%)	51 (37.5%)	19 (14%)
Closed suctioning	43 (31.7%)	29 (21.3%)	64 (47%)
Open suctioning	44 (30.2%)	51 (37.5%)	44 (32.3%)
IPPB	32 (23.5%)	33 (24.3%)	71 (52.2%)
Acapella	45 (33.1%)	48 (35.3%)	43 (31.6%)
Flutter	43 (31.6%)	49 (36%)	44 (32.4%)
Passive range of motion	62 (45.6%)	51 (37.5%)	23 (16.9%)
Active-assisted range of motion	85 (62.5%)	40 (29.4%)	11 (8.1%)
Active range of motion	89 (65.5%)	29 (21.3%)	18 (13.3%)
Balance training	36 (26.5%)	43 (31.6%)	57 (41.9%)
Ambulation	40 (29.4%)	59 (43.4%)	37 (27.2%)

**Table 5:** Frequency of different treatment methods used in patients presenting with saturation: high FiO_2_ and PEEP to maintain oxygenation; symptoms: altered mental status; signs of ARDS (mild to severe); HD CT scan: >15; oxygen support: MV or noninvasive ventilation (NIV).

	Always/frequently	Sometimes	Seldom/never
Breathing exercise	65 (47.7%)	31 (22.8%)	40 (29.4%)
Relaxation exercise	74 (54.4%)	40 (29.4%)	22 (16.2%)
Thoracic expansion exercise	53 (38.9%)	36 (26.5%)	47 (34.5%)
CARP protocol	62 (45.6%)	32 (23.5%)	42 (30.9%)
Prone positioning	80 (58.8%)	37 (27.2%)	19 (13.9%)
Postural drainage	99 (72.8%)	23 (16.9%)	14 (10.3%)
Incentive spirometry	47 (35.3%)	30 (22.1%)	59 (43.4%)
ACBT-FET	48 (35.3%)	34 (25%)	54 (39.7%)
Mechanical insufflation-exsufflation	50 (36.8%)	44 (32.4%)	42 (30.8%)
Percussion	88 (64.8%)	34 (25%)	14 (10.2%)
Vibration	84 (61.7%)	37 (27.2%)	15 (11%)
Closed suctioning	86 (63.3%)	33 (24.3%)	17 (12.5%)
Open suctioning	58 (42.7%)	48 (35.3%)	30 (22.1%)
IPPB	41 (30.1%)	28 (20.6%)	67 (49.3%)
Acapella	36 (26.4%)	25 (18.4%)	75 (55.2%)
Flutter	38 (27.9%)	23 (16.9%)	75 (55.2%)
Passive range of motion	85 (62.5%)	41 (30.1%)	10 (7.4%)
Active-assisted range of motion	77 (56.7%)	44 (32.4%)	15 (11%)
Active range of motion	65 (47.8%)	46 (33.8%)	25 (18.4%)
Balance training	29 (21.3%)	26 (19.1%)	81 (59.6%)
Ambulation	37 (27.2%)	26 (19.1%)	73 (53.7%)

**Table 6:** Frequency of different treatment methods used in patients presenting with saturation: needs high FiO_2_ and PEEP to maintain oxygenation; symptoms: ARDS, multiorgan failure, sepsis, and shock; oxygen support: MV or with ECMO.

	Always/frequently	Sometimes	Seldom/never
Breathing exercise	55 (40.5%)	25 (18.4%)	56 (41.2%)
Relaxation exercise	61 (44.9%)	35 (25.7%)	40 (29.4%)
Thoracic expansion exercise	47 (34.6%)	28 (20.6%)	61 (44.9%)
CARP protocol	51 (37.5%)	35 (25.7%)	50 (36.8%)
Prone positioning	71 (52.1%)	41 (30.1%)	24 (17.6%)
Postural drainage	88 (64.6%)	32 (23.5%)	16 (11.7%)
Incentive spirometry	38 (27.9%)	34 (25%)	64 (47%)
ACBT-FET	38 (27.9%)	30 (22.1%)	68 (50%)
Mechanical insufflation-exsufflation	48 (35.3%)	43 (31.6%)	45 (33%)
Percussion	87 (63.9%)	29 (21.3%)	20 (14.7%)
Vibration	84 (61.7%)	37 (27.2%)	15 (11%)
Closed suctioning	51 (58%)	35 (25.7%)	22 (16.1%)
Open suctioning	57 (42%)	45 (33.1%)	34 (25%)
IPPB	34 (24.9%)	36 (26.5%)	66 (48.5%)
Acapella	29 (21.3%)	35 (25.7%)	72 (52.9%)
Flutter	30 (22.1%)	34 (25%)	72 (52.9%)
Passive range of motion	76 (55.8%)	48 (35.2%)	12 (8.8%)
Active-assisted range of motion	66 (48.6%)	46 (33.8%)	24 (17.6%)
Active range of motion	52 (38.3%)	53 (38.9%)	31 (22.8%)
Balance training	25 (18.4%)	26 (19.1%)	85 (62.4%)
Ambulation	26 (19.1%)	30 (22.1%)	80 (58.8%)

**Table 7:** Association of different characteristics with knowledge, attitude, and practice.

Characteristics/*N*	Knowledge				Attitude				Practice			
	Mean	SD	*F*	*P*value	Mean	SD	*F*	*P*value	Mean	SD	*F*	*P*value
Qualification			19.94	≤0.01			1.434	0.242			4.607	0.012
BPT (69)	3.173	1.5714			2.869	0.9688			8.362	2.0144		
MPT (62)	4.371	1.1767			3.129	0.8774			9.387	1.8585		
Ph.D. (5)	6.400	2.0736			3.200	0.4472			9.000	1.5811		
Total (136)	3.838	1.6111			3.000	0.9189			8.852	1.9833		
Designation			6.931	≤0.01			0.683	0.564			1.930	0.128
Assoc. prof/asssit. prof/consultant physio (21)	4.904	1.5461			3.142	0.6546			9.047	2.2688		
PG (36)	3.861	1.4765			3.083	0.8742			9.194	1.8642		
Physiotherapist/lecturer (63)	3.777	1.5600			2.968	0.9994			8.857	1.8824		
Intern (16)	2.625	1.3601			2.750	1.000			7.812	2.0726		
Total (136)	3.838	1.6111			3.000	0.9189			8.852	1.9833		
Age			8.345	≤0.01			0.981	0.378			3.905	0.022
21–30 years (122)	3.680	1.4952			2.983	0.8905			8.737	1.9825		
31–40 years (8)	4.500	2.2038			2.875	1.356			9.000	1.6903		
>40 years (6)	6.166	1.1690			3.500	0.8366			11.00	1.0954		
Total (136)	3.838	1.6111			3.000	0.9189			8.852	1.9833		
Place of work (state)			0.786	0.504			1.688	0.173			1.045	0.375
Northern state (9)	3.3333	1.5811			3.000	0.7071			8.111	2.57121		
Western state (10)	4.1000	1.5238			2.500	1.269			9.000	1.76383		
Eastern state (8)	3.2500	1.3887			2.625	1.187			8.000	1.41421		
Southern state (109)	3.8991	1.6383			3.073	0.8682			8.963	1.98105		
Total	3.8382	1.6111			3.000	0.9189			8.852	1.98337		
Duration of work			10.077	≤0.01			1.297	0.274			3.944	0.005
1–3 months (52)	3.6346	1.5085			2.903	0.9130			8.557	1.88298		
3–5 months (23)	3.9565	1.2960			2.913	0.9001			9.043	1.87030		
6–12 months (13)	4.6923	1.0315			3.153	0.9871			9.538	1.98391		
<1 months (27)	2.7037	1.4362			2.888	1.012			8.037	2.06587		
>1 years (21)	5.1429	1.5583			3.381	0.7400			10.00	1.70294		
Total	3.8382	1.6111			3.000	0.9189			8.852	1.98337		

## Data Availability

The data used in this study can be made available on requesting the corresponding author.

## Supplementary Materials

Supplementary Materials This questionnaire consists of the demographics of the respondents and 23 questions divided into three sections. Section A includes seven questions aimed to assess physiotherapists' knowledge regarding physiotherapy guidelines, Section B includes four questions regarding physiotherapists' attitudes toward patient care, and Section C has twelve questions focused on assessing their practice patterns in COVID-19 ICUs.
