# Supplementary material for: Knowledge, Attitude, and Practice of Physiotherapists in COVID-19 ICUs: A National Survey
Source: ScientificWorldJournal. 2024 Jan 17;2024:9918558. doi: 10.1155/2024/9918558 (PMC10807948; doi:10.1155/2024/9918558)
Supplement: Supplementary Materials — This questionnaire consists of the demographics of the respondents and 23 questions divided into three sections. Section A includes seven questions aimed to assess physiotherapists' knowledge regarding physiotherapy guidelines, Section B includes four questions regarding physiotherapists' attitudes toward patient care, and Section C has twelve questions focused on assessing their practice patterns in COVID-19 ICUs. [file 9918558.f1.docx]

**Appendix 1**

Demographics;

1. What is your highest level of education?

- BPT
- MPT
- Ph D

1. Age: _______
2. What is your gender?

- Male
- Female
- Others

1. In which state of India do you work?

________________

1. Designation at the workplace?

________________

1. How long have you worked in Covid ICU?

- Less than one month
- 1-3 months
- 3-6 months
- 6-12 months
- More than one year

SECTION A

1. Did the physiotherapists, including you, receive any special training before you started working in Covid ICU?

- Yes
- No
- If yes, what type of training ______________

1. Are you aware of the technique and sequence of donning and doffing PPE?

- Yes
- No

1. Are you familiar with COVID awake repositioning proning (CARP) protocol?

- I know this really well
- I know this
- I have heard of this
- I don’t think I have heard this

1. Do you think prone positioning for patients with severe ARDS is applicable in Covid patients too?

- Yes
- No

If yes, then what is the recommended duration per day

- 12- 16 hours
- 24 hours
- Not more than 2 hours
- Not sure
- Any other ________

1. The following are the criteria for closed monitoring during treatment.

- Fall of oxygen saturation more than 3% or saturation of < 96% at rest.
- Fall of oxygen saturation more than 10% or saturation of <85% at rest.
- None of the above.
- Not sure

1. Which of the following category of patients are indicated for Physiotherapy?

- Mild symptoms such as dry cough and fever; without significant respiratory compromise, Low-level oxygen requirement (e.g., oxygen flow ≤ 5L/min for spo2 ≥ 90%), patient able to cough effectively
- Mild symptoms and/or pneumonia AND coexisting respiratory or neuromuscular comorbidity. (E.g., cystic fibrosis, neuromuscular comorbidity, Chronic Obstructive Pulmonary Disease, etc.) AND current or anticipated difficulties with secretion clearance.
- Mild symptoms and/or pneumonia AND evidence of exudative consolidation with difficulty clearing or inability to clear secretion inadequately.
- Severe symptoms suggesting pneumonia/ lower respiratory tract infection. E.g., increasing oxygen requirements, fever, difficulty breathing, frequent/severe/productive cough, chest x-ray/ CT scan/ lung ultrasound shows changes consistent with consolidation.
- Any patient at significant risk of developing or with mild evidence of significant functional limitation. E.g., multiple comorbidities, a patient at risk of ICU acquired weakness.

1. Do you think that strengthening skeletal muscles and recovery of ADL should be contraindicated in patients admitted in Covid ICU, as they can increase the load on the respiratory system and increase the risk of distress?

- Yes
- No
- Not sure

SECTION B

1. Please assume for a moment about exposure to the therapist and answer the question.

Physiotherapy service is essential in Covid-ICU?

- Strongly Agree
- Agree
- Neutral
- Disagree
- Strongly Disagree

1. Which phase of covid do you think Physiotherapy should be initiated?

- Acute/mild
- Critical
- Post-acute and discharge
- All of the above

1. Do you follow a multidisciplinary approach?

- Always
- Frequently
- Sometimes
- Seldom
- Never

1. Is the physiotherapist’s opinion taken before shifting the patient out from Covid ICU?

- Always
- Frequently
- Sometimes
- Seldom
- Never

SECTION C

1. Did you have an opportunity to monitor and record vital details pre and post-treatment?

- Always
- Frequently
- Sometimes
- Seldom
- Never

1. Was prone positioning a common strategy used to improve saturation at your setup?

- Always
- Frequently
- Sometimes
- Seldom
- Never

1. Did you have an opportunity topractice or recommend a frequent change of position?

- Always
- Frequently
- Sometimes
- Seldom
- Never

1. Were you involved in airway suctioning for bronchial hygiene of COVID patients?

Open suction

- Always
- Frequently
- Sometimes
- Seldom
- Never

Close suction

- Always
- Frequently
- Sometimes
- Seldom
- Never

1. Did you have an opportunity to synchronize timing for bronchial hygiene therapy or airway clearance technique with nebulization?

- Always
- Frequently
- Sometimes
- Seldom
- Never

1. Did you have an opportunity to use the Positive Expiratory Pressure (PEP) device?

- Always
- Frequency
- Sometimes
- Seldom
- Never

Mention the frequently used PEP devices__________

1. Do you prefer to provide early mobilization in mechanically ventilated patients?

- Always
- Frequency
- Sometimes
- Seldom
- Never

1. Did you have an opportunity to ambulate stable patients within ICU?

- Always
- Frequency
- Sometimes
- Seldom
- Never

1. In the table given below, mark all the treatments that you would provide a patient presenting with:

Saturation: Mild or No change at rest (SpO2 of 92-94%)

Symptoms: None

HD CT score: <8

Oxygen support: Low flow oxygen or Room air/ home isolation

|  | Always | frequently | sometimes | Seldom | Never |
| --- | --- | --- | --- | --- | --- |
| Breathing exercise |  |  |  |  |  |
| Relaxation exercises |  |  |  |  |  |
| Thoracic Expansion Exercise |  |  |  |  |  |
| CARP protocol |  |  |  |  |  |
| Prone positioning |  |  |  |  |  |
| Postural drainage |  |  |  |  |  |
| Incentive spirometry |  |  |  |  |  |
| ACBT  FET |  |  |  |  |  |
| Mechanical insufflation exsufflation |  |  |  |  |  |
| Percussion |  |  |  |  |  |
| Vibration |  |  |  |  |  |
| Closed suctioning |  |  |  |  |  |
| Open suctioning |  |  |  |  |  |
| IPPB |  |  |  |  |  |
| Acapella |  |  |  |  |  |
| flutter |  |  |  |  |  |
| Passive Range of motion |  |  |  |  |  |
| Active assisted Range of motion |  |  |  |  |  |
| Active Range of motion |  |  |  |  |  |
| Balance training |  |  |  |  |  |
| Ambulation |  |  |  |  |  |

Others ________________

1. In the table given below, mark all the treatments that you would provide a patient presenting with:

Saturation: Drop in SpO2 during activity

Symptoms: fever, cough, breathlessness

HD CT scan: 9-19

Oxygen support: High flow oxygen (HFO) system/ venturi mask

|  | Always | frequently | sometimes | Seldom | Never |
| --- | --- | --- | --- | --- | --- |
| Breathing exercise |  |  |  |  |  |
| Relaxation exercises |  |  |  |  |  |
| Thoracic Expansion Exercise |  |  |  |  |  |
| CARP protocol |  |  |  |  |  |
| Prone positioning |  |  |  |  |  |
| Postural drainage |  |  |  |  |  |
| Incentive spirometry |  |  |  |  |  |
| ACBT  FET |  |  |  |  |  |
| Mechanical insufflation exsufflation |  |  |  |  |  |
| Percussion |  |  |  |  |  |
| Vibration |  |  |  |  |  |
| Closed suctioning |  |  |  |  |  |
| Open suctioning |  |  |  |  |  |
| IPPB |  |  |  |  |  |
| Acapella |  |  |  |  |  |
| flutter |  |  |  |  |  |
| Passive Range of motion |  |  |  |  |  |
| Active assisted Range of motion |  |  |  |  |  |
| Active Range of motion |  |  |  |  |  |
| Balance training |  |  |  |  |  |
| Ambulation |  |  |  |  |  |

Others ___________________

1. In the table given below, mark all the treatments that you would provide a patient presenting with:

Saturation: High FiO2 and PEEP to maintain oxygenation

Symptoms: Altered mental status Signs of ARDS (mild to Severe)

HD CT scan: >15

Oxygen support: Mechanical ventilator (MV) or non-invasiveventilation (NIV)

|  | Always | frequently | sometimes | Seldom | Never |
| --- | --- | --- | --- | --- | --- |
| Breathing exercise |  |  |  |  |  |
| Relaxation exercises |  |  |  |  |  |
| Thoracic Expansion Exercise |  |  |  |  |  |
| CARP protocol |  |  |  |  |  |
| Prone positioning |  |  |  |  |  |
| Postural drainage |  |  |  |  |  |
| Incentive spirometry |  |  |  |  |  |
| ACBT  FET |  |  |  |  |  |
| Mechanical insufflation exsufflation |  |  |  |  |  |
| Percussion |  |  |  |  |  |
| Vibration |  |  |  |  |  |
| Closed suctioning |  |  |  |  |  |
| Open suctioning |  |  |  |  |  |
| IPPB |  |  |  |  |  |
| Acapella |  |  |  |  |  |
| flutter |  |  |  |  |  |
| Passive Range of motion |  |  |  |  |  |
| Active assisted Range of motion |  |  |  |  |  |
| Active Range of motion |  |  |  |  |  |
| Balance training |  |  |  |  |  |
| Ambulation |  |  |  |  |  |

Others ____________________

1. In the table given below, mark all the treatments that you would provide a patient presenting with:

Saturation: Needs High FiO2 & PEEP to maintain oxygenation

Symptoms: ARDS, multiorgan failure, sepsis, shock

Oxygen support: Mechanical ventilator or on Extra Corporeal Membrane oxygenation (ECMO)

|  | Always | frequently | sometimes | Seldom | Never |
| --- | --- | --- | --- | --- | --- |
| Breathing exercise |  |  |  |  |  |
| Relaxation exercises |  |  |  |  |  |
| Thoracic Expansion Exercise |  |  |  |  |  |
| CARP protocol |  |  |  |  |  |
| Prone positioning |  |  |  |  |  |
| Postural drainage |  |  |  |  |  |
| Incentive spirometry |  |  |  |  |  |
| ACBT  FET |  |  |  |  |  |
| Mechanical insufflation exsufflation |  |  |  |  |  |
| Percussion |  |  |  |  |  |
| Vibration |  |  |  |  |  |
| Closed suctioning |  |  |  |  |  |
| Open suctioning |  |  |  |  |  |
| IPPB |  |  |  |  |  |
| Acapella |  |  |  |  |  |
| flutter |  |  |  |  |  |
| Passive Range of motion |  |  |  |  |  |
| Active assisted Range of motion |  |  |  |  |  |
| Active Range of motion |  |  |  |  |  |
| Balance training |  |  |  |  |  |
| Ambulation |  |  |  |  |  |

Others ____________________
